# Screening for Microsatellite Instability Identifies Frequent 3′-Untranslated Region Mutation of the RB1-Inducible Coiled-Coil 1 Gene in Colon Tumors

**DOI:** 10.1371/journal.pone.0007715

**Published:** 2009-11-02

**Authors:** Bogdan C. Paun, Yulan Cheng, Barbara A. Leggett, Joanne Young, Stephen J. Meltzer, Yuriko Mori

**Affiliations:** 1 Division of Gastroenterology, Department of Medicine, Johns Hopkins University School of Medicine, Baltimore, Maryland, United States of America; 2 Division of Gastroenterology, Department of Oncology, Johns Hopkins University School of Medicine, Baltimore, Maryland, United States of America; 3 Conjoint Gastroenterology Lab, Royal Brisbane Hospital Foundation, Clinical Research Centre, Bancroft Centre, Herston, Queensland, Australia; 4 Familial Cancer Laboratory, Queensland Institute of Medical Research, Herston, Queensland, Australia; Duke-NUS Graduate Medical School, Singapore

## Abstract

**Background:**

Coding region microsatellite instability (MSI) results in loss of gene products and promotion of microsatellite-unstable (MSI-H) carcinogenesis. Recent studies have indicated that MSI within 3′-untranslated regions (3′UTRs) may post-transcriptionally dysregulate gene products. Within this context, we conducted a broad mutational survey of 42 short 3′UTR microsatellites (MSs) in 45 MSI-H colorectal tumors and their corresponding normal colonic mucosae.

**Methodology/Principal Findings:**

In order to estimate the overall susceptibility of MSs to MSI in MSI-H tumors, the observed MSI frequency of each MS was correlated with its length, interspecies sequence conservation level, and distance from some genetic elements (i.e., stop codon, polyA signal, and microRNA binding sites). All MSs were stable in normal colonic mucosae. The MSI frequency at each MS in MSI-H tumors was independent of sequence conservation level and distance from other genetic elements. In contrast, MS length correlated significantly with MSI frequency in MSI-H tumors (r = 0.86, p = 7.2×10^−13^). 3′UTR MSs demonstrated MSI frequencies in MSI-H tumors higher than the 99% upper limit predicted by MS length for *RB1-inducible coiled-coil 1*(*RB1CC1*, mutation frequency 68.4%), *NUAK family SNF1-like kinase 1*(*NUAK1*, 31.0%), and *Rtf1, Paf1/RNA polymerase II complex component, homolog* (*RTF1*, 25.0%). An *in silico* prediction of RNA structure alterations was conducted for these MSI events to gauge their likelihood of affecting post-transcriptional regulation. *RB1CC1* mutant was predicted to lose a microRNA-accessible loop structure at a putative binding site for the tumor-suppressive microRNA, miR-138. In contrast, the predicted 3′UTR structural change was minimal for *NUAK1-* and *RTF1* mutants. Notably, real-time quantitative RT-PCR analysis revealed significant *RB1CC1* mRNA overexpression *vs.* normal colonic mucosae in MSI-H cancers manifesting *RB1CC1* 3′UTR MSI (9.0-fold; p = 3.6×10^−4^).

**Conclusions:**

This mutational survey of well-characterized short 3′UTR MSs confirms that MSI incidence in MSI-H colorectal tumors correlates with MS length, but not with sequence conservation level or distance from other genetic elements. This study also identifies *RB1CC1* as a novel target of frequent mutation and aberrant upregulation in MSI-H colorectal tumors. The predicted loss of a microRNA-accessible structure in mutant *RB1CC1* RNA fits the hypothesis that 3′UTR MSI involves in aberrant *RB1CC1* posttranscriptional upregulation. Further direct assessments are indicated to investigate this possibility.

## Introduction

High-level microsatellite instability (MSI-H) is the molecular hallmark of a subset of colorectal cancers (CRCs) which carry defects in DNA mismatch repair (MMR). MSI is defined as nucleotide length abnormalities occurring within short DNA sequences consisting of iterated oligonucleotide units (microsatellites), and is widespread throughout the genomes of MSI-H CRC. MSI exerts its tumorigenic effects when it occurs within protein coding regions thereby disabling tumor suppressor genes in MSI-H CRCs via frameshift mutation [1]. The genome-wide distributions of these coding MSI events have been studied extensively in different tumor types by several groups, including our own [2], [3], [4].

MSI also occurs within the 3′-untranslated regions (3′UTRs) of genes. Recent advances in RNA research have revealed that the 3′UTR plays a prominent role in regulating the stability, subcellular localization, and translation of its parent mRNA via sequence-specific interactions with trans-acting factors including small RNAs and proteins [5]. Mutations within the 3′UTR can affect gene activity if they alter RNA sequence or structure relevant to these interactions. Several 3′UTR point mutations have been linked to the risk of developing cancer in humans [6], [7], [8]. Recent reports have also shown that deleterious mutations at two 3′UTR 8-mer mononucleotide repeats destabilize the mRNAs in which these mutations occur [9], [10]. Taken together, 3′UTR MSI events are likely to be important in defining MSI-H cancer phenotypes, analogous to coding region MSI events. However, in contrast to coding region MSI, information on 3′UTR MSI in MSI-H cancers is limited.

One challenge inherent in MSI profiling is to discriminate mutations that contribute to carcinogenesis from innocuous “bystander” or “passenger” mutations [11]. A study of long (15- to 32-mer) 3′UTR mononucleotide repeats revealed that these loci are sometimes polymorphic in MMR-proficient cells, but almost always unstable in MMR-deficient cells [12]. Thus, we expect that profiling shorter microsatellites would be more fruitful in identifying MSIs that were functionally relevant to MSI-H carcinogenesis.

In the current study, we performed broad mutational profiling of 42 short 3′UTR microsatellites (8–14 bases in length) in 45 primary MSI-H colorectal tumors. We also assessed the correlation between MSI prevalence and microsatellite attributes, as well as the impact of MSI upon RNA secondary structure. We utilized the results of these assessments as the basis to discriminate carcinogenic MSI from likely passenger MSI exerting no beneficial effect upon tumor cell survival.

## Materials and Methods

### Identification, enrollment, and annotation of 3′UTR microsatellite loci

3′UTR mononucleotide repeats were identified during our previous genome-wide coding microsatellite search [2]. In this genome-wide search, 44 loci were initially erroneously classified as coding region loci by UniGene, but ultimately reclassified correctly as 3′UTR loci after our own individual inspection. In the current study, 42 of these 44 3′UTR mononucleotide repeats (8–14 bases) were enrolled without regard to their putative functions (two loci were dropped because of unsuccessful establishment of microsatellite-specific PCR assays). The nucleotide positions of flanking polyA sites and predicted microRNA (miR) binding sites, along with the PhastCons basewise conservation score (0 to 1, 1 for nucleotides in the most conserved sequence contexts among 31 placental mammals) for each microsatellite, were obtained from the PolyA_DB table under poly(A) track, the TargetScan table under TS miR sites track, and PhastCons44Placental table under Conservation track, respectively, within the UCSC Table Browser [13], [14], [15], [16]. Data on AU-rich elements (ARE) were obtained from the Integrated ARED database [17]. Putative miR binding sites were listed only when their presence was also supported by an independent algorithm, miRanda [18]. Descriptive data for each microsatellite are shown in **Table S1**.

### Patients and sample DNA preparation

We obtained 44 MSI-H CRCs (nine HNPCC-associated and 35 sporadic), one MSI-H colon adenoma (HNPCC), and their corresponding normal colonic mucosal tissues from patients who underwent surgery at the Royal Brisbane Hospital Foundation Clinical Research Center. Two of the nine HNPCC-associated CRCs originated from a single patient in a synchronous manner. Patient enrollment and subsequent study were conducted according to protocols approved by the Institutional Review Board at Johns Hopkins University and the Royal Brisbane Hospital Foundation. Written informed consent was obtained from all patients. Genomic DNA was extracted from tissues that had been snap-frozen after mucosal layer enrichment by macroscopic dissection. The MSI-H status of each tumor was validated by the presence of MSI at two or more of five consensus loci (BAT25, BAT26, D2S123, D5S346 and D17S250) [19]. Descriptive data for each tumor are shown in **Table 1**. The significantly younger age of HNPCC patients relative to sporadic cases is consistent with the young-onset nature of the condition [19].

**Table 1:** Case demographic data.

Category	All (n = 45)	HNPCC (n = 10)	Sporadic (n = 35)	p-value (test)
**Gender**				
M	17	5	12	0.47 (Fisher's exact test)
F	28	5	23	
**Age**				
mean (*SD)	65.9 (13.0)	48.3 (10.5)	70.9 (8.5)	1.1×10^−8^ (Student's t-test)
**Duke's stage**				
A	4	0	4	0.029 (Kruskal-Wallis test)
B	28	6	22	
C	8	1	7	
D	2	1	1	
unknown	3	2	1	
**Differentiation**				
well	2	0	2	0.029 (Kruskal-Wallis test)
moderate	15	3	12	
poor	15	3	12	
unknown	13	4	9	
**Location**				
L	6	2	4	0.60 (Fisher's exact test)
R	39	8	31	

All cases were Caucasian. HNPCC patients included one adenoma. P-values were calculated for the comparison of tumors from HNPCC patients versus sporadic cases.

*SD: standard deviation.

### PCR fragment length polymorphism analysis

PCR fragment length polymorphism analysis was used for MSI status assessment, as described previously [2]. PCR primer sequences are listed in **Table S1**. Instability at each locus was measured by two variables: %MSI and mean shift. %MSI represented the prevalence of specimens demonstrating MSI in all informative specimens at each locus. Mean shift constituted the mean absolute microsatellite length change per specimen in all informative specimens at each locus. Monomorphic, quasi-monomorphic, and polymorphic loci were defined as loci whose mean shift (bases/specimen) was equal to 0, greater than 0 but less than 1, and equal to 1 or greater, respectively, as described previously [12]. Variants present in normal tissues were excluded from the calculation of somatic %MSI and mean shift for tumors.

### Statistic: regression analysis assessing the dependency of %MSI upon microsatellite length

For this regression analysis, only data on 8- to 11-mer 3′UTR microsatellites were used as input: 12 to 14-mers were excluded because only single-locus data was available for each length. Based upon a meta-analysis of coding and noncoding microsatellites in MSI-H CRCs, the correlation between %MSI and microsatellite length followed a two-stage or sigmoid pattern: %MSI increased quasi-exponentially in the short microsatellite length range, then formed a plateau when %MSI approached 100% [20]. Based upon a previous study of 15- to 28-mer 3′UTR microsatellites that were stable in the germline, mean shift in MSI-H CRCs was estimated to reach 1 when microsatellite length was between 13 and 14 bases [12]. Our data on 8- to 11-mer microsatellites was concluded to represent mutational profiles in the quasi-exponential stage. Therefore, we employed the following exponential growth model for estimating the dependency of %MSI on microsatellite length:
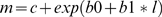
where *m* and *l* are expected %MSI and microsatellite length, respectively, while *c*, *b0*, and *b1* are parameters. Estimation of the optimal parameter values to fit the actual data was conducted based upon the Levenberg–Marquardt algorithm [21]. In calculating the optimal parameter values, we also assumed that mutational prevalence was 0 when microsatellite length was 1. As a measure of the goodness of fit of the resulting model, the correlation coefficient between observed and expected values was calculated for the microsatellite length range in which this model could be adequately representative (*i.e.*, the range where expected mutation prevalence did not reach 100%). Based upon this regression model, 95% confidence interval and 99% prediction interval were calculated for the expected %MSI according to microsatellite length.

### Prediction of mRNA secondary structure

RNA secondary structure and free energy for the wild type and the dominant mutant at each locus were calculated by utilizing pknotsRG-enf script [22]. RNA sequences containing the microsatellite and 200 bases of its 5′ and 3′ flanking regions were used as input.

### Quantitative RT-PCR

Total RNA extraction, genomic DNA degradation, and random hexamer-based cDNA synthesis for five normal colonic mucosae and eight MSI-H primary CRCs were performed using an RNeasy kit (Qiagen) and SuperScript III reverse transcriptase (Invitrogen). Real-time quantitative RT-PCR assays were performed on an iQ5 PCR machine (Bio-Rad) using iQ SYBR Green Supermix (Bio-Rad) according to the manufacturer's protocol. *Beta*-*actin* was analyzed for normalization of data, as described previously [23]. Lack of non-specific PCR products was verified by melting curve analysis of all PCR products. The *RB1CC1* amplicon (annealing temperature 56 degrees C) used primer sequences 5′-TGACAATATACTTCACTGGT-3′ (forward) and 5′-GCAATTAATATGGCTCATCA-3′ (reverse), and the *beta-actin* amplicon (annealing temperature 60 degrees C) used primer sequences 5′-ACCATGGATGATGATATCGCC-3′ (forward) and 5′-GCCTTGCACATGCCGG-3′ (reverse).

## Results

Somatic and germline MSI profiles for 42 microsatellites (8 to 14-mers) located within 3′UTRs were assessed in 45 primary MSI-H colorectal tumors and their corresponding normal colonic mucosae obtained from 44 patients, respectively. These 44 cases consisted of 35 sporadic CRC patients and nine HNPCC patients; one HNPCC subject had two synchronous CRCs. MSI prevalence at each 3′UTR locus was correlated with microsatellite length, distance from stop codon and polyA site, and local interspecies conservation level to determine factors that could control instability of 3′UTR microsatellites.

### 3′UTR microsatellites are stable in germline cells

**Figure 1:**
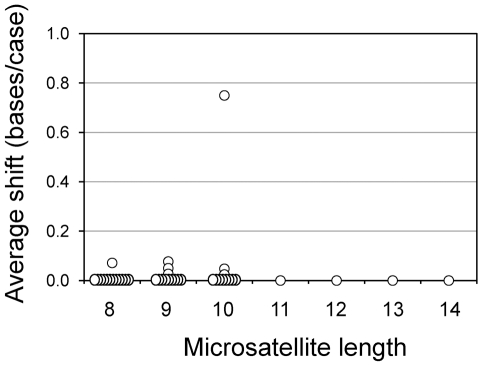
Germline instability profile of the 42 3′UTR microsatellites *vs.* microsatellite length. This scatterplot shows the averaged change in microsatellite length (mean base shift, *Y-axis*) in normal colonic tissues of the 45 MSI-H colorectal tumor patients for each of the 42 microsatellite loci and its association with microsatellite length (*X-axis*). Each datapoint represents a 3′UTR microsatellite. There was no significant correlation between microsatellite length and mean base shift.

In order to determine variability at each locus in MMR-proficient cells, we analyzed germline length polymorphisms of the 42 3′UTR microsatellites in normal colonic tissues obtained from the 44 MSI-H colorectal tumor patients. All 42 microsatellites were stable: 35 loci were monomorphic, while seven loci (one of thirteen 8-mers, two of twelve 9-mers, and three of eleven 10-mers) were quasi-monomorphic (**Figure 1**). The prevalence of stable (i.e., monomorphic and quasi-monomorphic) loci in our 8- to 14-mer group (42 of 42 loci, 100%) was significantly greater relative to the reported prevalence in longer microsatellite groups: eight (80%) of ten loci in a 15- to 20-mer group (Fisher's exact test p-value 0.03), and five (29.4%) of 17 loci in the 21- to 32-mer group (Fisher's exact test p-value, 5.0×10^−9^) [12]. No significant correlation was detected between mean shift and interspecies sequence conservation level or distance from stop codon or polyA signal for each microsatellite (|r|<0.1). All seven quasi-monomorphic loci possessed only one type of variant consisting of one-base insertion or deletion. Mean shifts for these quasi-monomorphic loci were less than 0.1 except for one locus: *NUAK family SNF1-like kinase 1* (*NUAK1*; mean shift 0.75). Prevalence of germline variants for these seven monomorphic loci was not significantly different between sporadic CRC and HNPCC patients (**Table S2**).

### 3UTR microsatellite mutation status in MSI-H tumors is dependent on microsatellite length

**Figure 2:**
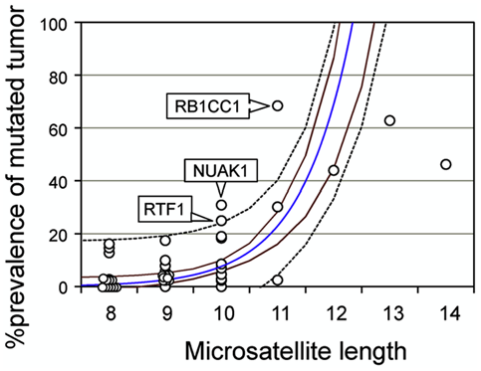
Somatic mutational profile of the 42 3′UTR microsatellites *vs.* microsatellite length. This scatterplot shows the prevalence of mutated tumors at each microsatellite (*Y-axis*) for the 45 MSI-H colorectal tumors and its significant association with microsatellite length (*X-axis*). Each datapoint represents a 3′UTR microsatellite locus. The *blue line* represents the fit curve calculated based upon non-linear regression analysis: the r- and the p-values were 0.86 and 7.2×10^−13^, respectively, for the correlation between observed and expected mutation prevalence. *Brown line* and *dotted black lines* signify 95% confidence limits and 99% prediction limits, respectively.

Somatic mutation status of the 42 3′UTR microsatellites was analyzed in all 45 primary MSI-H colorectal tumors (35 sporadic CRCs, nine HNPCC-CRCs, and one HNPCC-associated adenoma). Variants observed in germline cells were treated as wild-type. Eight loci were not mutated in any of the 45 tumors, while 34 loci showed varying mutation rates up to 68.4%. We then correlated this somatic mutational profile with microsatellite attributes. Similarly to germline variation, somatic mutation frequency did not correlate significantly with local interspecies conservation level or distance from stop codon or polyA site (|r|<0.1). However, somatic mutation prevalence did correlate significantly with microsatellite length. A regression analysis revealed that the %MSI fit an exponential growth curve dependent on microsatellite length (r = 0.86 and p = 7.2×10^−13^; **Figure 2**). The %MSI fit curve exceeded 100% at a microsatellite length of 13 bases, which was consistent with previously published data on longer 3′UTR microsatellites [12]. Additionally, somatic mutation frequency was significantly higher in loci with flanking putative miR binding sites than in those without flanking miR binding sites (21.8% vs. 8.7%; t-test p-value 0.02). However, loci with flanking miR binding sites tended to be longer than loci without flanking miR binding sites (9.9 bases vs. 9.1 bases; t-test p-value 0.15). No significant difference in mutation rate depending upon the presence of flanking putative miR binding sites was observed when loci of the same length were compared.

Three loci in our group demonstrated exceptionally high %MSI in MSI-H colorectal tumors that exceeded the 99% prediction limit for expected %MSI according to their lengths (**Figure 2**, **Table 2**, and **Table S1**). The T_11_ repeat in the *RB1-inducible coiled-coil 1*(*RB1CC1*) gene exhibited one- or two-base deletions in 26 (68.4%) of 38 informative cases, even though its corresponding upper 99% prediction limit was only 40.2%. Similarly, the A_10_ repeat in the *NUAK1* gene exhibited one- or two-base deletions in 13 (31.0%) of 42 cases. Furthermore, the T_10_ repeat in the *Rtf1, Paf1/RNA polymerase II complex component, homolog* (*RTF1*) gene exhibited one-base deletions or insertions in ten (25.0%) of 40 cases. The corresponding upper 99% prediction limit of these two repeats was 24.0%. Notably, *RB1CC1* biallelic mutation was significantly infrequent (two of 38 cases, 5.3%) relative to the expected frequency based upon mutant allele incidence (13.6%, five of 38 cases; Fisher's exact test p value, 0.01). Biallelic mutation in *NUAK1* (two of 42, 4.8%) and *RTF1* (zero of 41, 0%) did not significantly differ in prevalence from their expected frequencies (3.2% and 1.6% for *NUAK1* and *RTF1*, respectively). No statistically significant association was observed between the presence of these mutations and background disease, anatomical site within the colon, clinical stage, or degree of histological differentiation for the informative tumors (**Table S3**).

**Table 2:** Locus description and somatic mutational properties of three highly mutable 3′UTR microsatellites.

Category	RB1CC1	NUAK1	RTF1
Repeat	T_11_	A_10_	T_10_
Locus	8q11	12q23.3	15q15.1
Distance from stop codon (bases)	219	1964	2131
Distance from polyA signal (bases)	970	1471	741
*PhastCons score: mean (SD)	0.21 (0.22)	0.67 (0.32)	0.00 (0.00)
**Flanking miR binding sites	miR-138, miR-133	**−**	miR-26, miR-139
***Flanking ARE motifs	**+**	**+**	**−**
Total informative tumors (%)	38 (100.0)	42 (100.0)	40 (100.0)
Mutant tumors (%)	26 (68.4)	13 (31.0)	10 (25.0)
Wild type tumors (%)	12 (31.6)	29 (69.0)	30 (75.0)
Major mutant	T_10_	A_9_	T_9_
%Δ free energy of the major mutant	0	0	−0.1
Structure change of the major mutant	**+**	**−**	**+**

Locus information and overall mutation prevalence describes the genetic attributes and mutation status in MSI-H colon tumors for microsatellites located within the 3′UTRs of *RB1CC1*, *NUAK1*, and *RTF1*.

* *PhastCons score*: Mean and standard deviation of basewise microsatellite sequence conservation score among 31 placental mammals. The value closer to 1 indicates a high conservation level, and a large standard deviation indicates a change in conservation pattern within the microsatellite.

** *Flanking miR binding sites*: Putative miR binding sites predicted by TargetScan and miRanda scripts within 200 bases of the 3′ or 5′ flanking regions of the corresponding microsatellite.

*** Flanking *ARE motifs*: presence of AU-rich elements within the 3′UTR, according to the Integrated ARED database.

**Figure 3:**
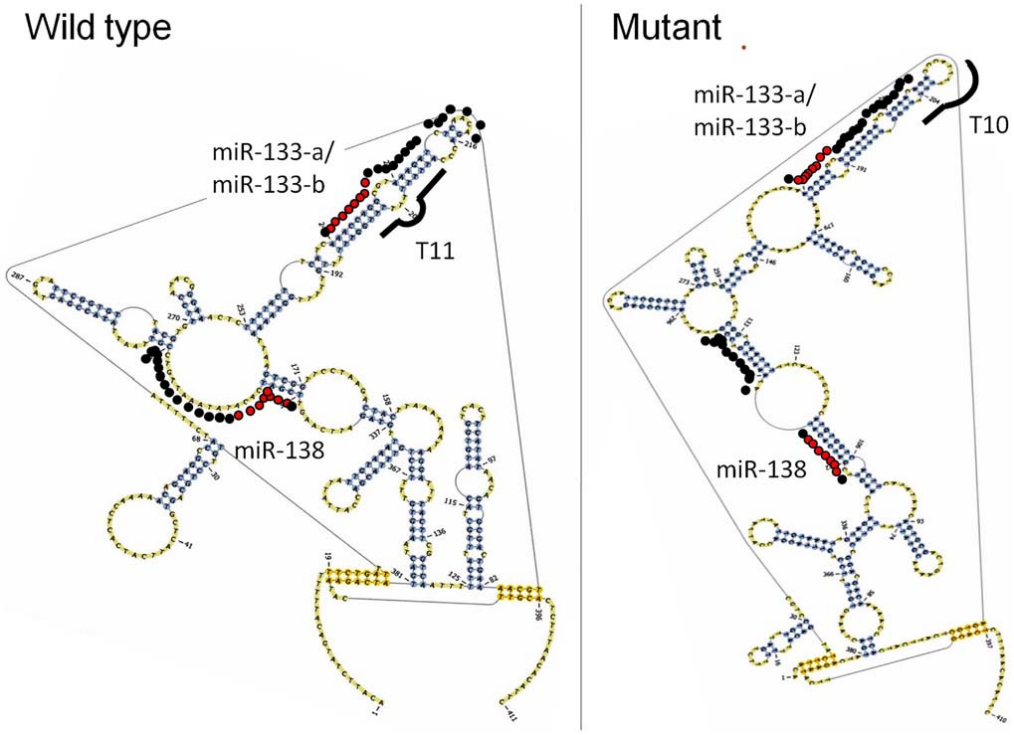
The impact of MSI upon 3′UTR mRNA secondary structure of the *RB1CC1* gene. This drawing illustrates the difference in predicted secondary mRNA structure of the *RB1CC1* 3′UTR containing putative microRNAs binding sites between wild type (T_11_) and the most prevalent microsatellite mutant (T_10_) observed in the MSI-H colorectal tumors. A *black line* represents the microsatellite. *Dots* represent putative microRNA binding sites (the 3′-complementary site is highlighted in *red*). Secondary structure was altered for both the miR-133-a/b and miR-138 binding sites in the T_10_ mutant. The miR-138 binding site structure demonstrated the greatest change caused by the microsatellite mutation: the mutant lost a large loop structure that covered the majority of both 5′ seed and 3′ complementary sites that had existed in the wild-type.

**Figure 4:**
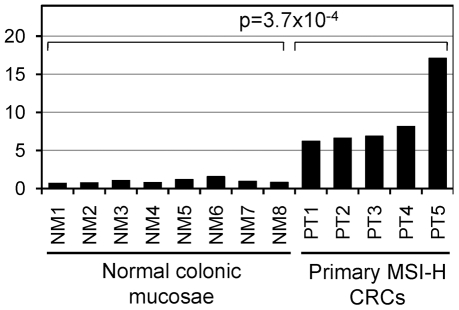
*RB1CC1* mRNA overexpression in primary MSI-H colon cancers. Quantitative RT-PCR data for RB1CC1 are shown in eight normal colonic mucosae (NMs) and five primary MSI-H CRCs (PTs). A ratio to the average of 8NMs is shown as the value representing the *beta-actin*-normalized *RB1CC1* mRNA expression in each sample. *RB1CC1* was significantly overexpressed in primary MSI-H CRCs relative to normal colonic mucosae (mean fold-change 9.0, p = 3.7×10^−4^) base deletion.

In order to make a preliminary assessment of the potential functional impact of 3′UTR MSI, we analyzed the predicted RNA secondary structure and free energy for the wild type and the most common mutant of the T_11_ repeat in *RB1CC1*, the A_10_ repeat in *NUAK1*, and the T_10_ repeat in *RTF1* (**Table 2**). The most common *RB1CC1* mutant, T_10_, greatly altered its predicted 3′UTR RNA secondary structure (**Figure 3**). This predicted *RB1CC1* structural change affected putative miR-133 and miR-138 binding sites flanking the microsatellite, and the miR-138 binding site structure demonstrated the greatest change caused by the mutation. The *RB1CC1* mutant lost a large loop structure that covered the majority of both 5′ seed and 3′ complementary sites that had existed in the wild-type RNA. Interestingly, we observed significant *RB1CC1* mRNA overexpression in primary MSI-H CRCs carrying *RB1CC1* mutant alleles relative to normal colonic mucosae (mean fold-change 9.0, p = 3.7×10^−4^, **Figure 4**). The most common *RTF1* mutant, T_9_, altered both free energy and predicted RNA structure (data not shown). However, the affected *RTF1* 3′UTR region was small (up to 50 bases 5′ and 3′ of the microsatellite) and did not contain any putative 3′UTR regulatory motifs. Consequently, this mutation was unlikely to result in sizable functional effects. The most common *NUAK1* mutant, A_9_, did not affect either RNA secondary structure or free energy.

## Discussion

The current broad mutational profiling of 42 short (8–14 base) 3′UTR microsatellites revealed that somatic microsatellite mutation frequency in 45 MSI-H colon tumors correlated significantly with microsatellite length, which was consistent to a previous meta-analysis on coding and noncoding microsatellites [20]. The overall mutation frequency was higher in this meta-analysis than the current study. Mutation frequency in the meta-analysis might have been high, in part, due to the publication bias against negative mutational data, the employment of functional knowledge-based subject locus selection in many reports, and absence of consideration to germline polymorphism level in many reports. Germline variation prevalence was not significantly dependent on microsatellite length within our short microsatellite group; however, a significant microsatellite length dependency of germline variation prevalence was observed when our data were compared to previously studied groups of longer (15–20 and 21–28 base) 3′UTR microsatellites [12]. In yeast, repeat number and repeat unit size dictate the susceptibility to DNA strand slippage and microsatellite instability [24], [25]. Thus, our observations confirm that susceptibility to DNA strand slippage is the determinant of length polymorphism in the majority of 3′UTR microsatellites in both MMR-deficient and -proficient settings in humans.

In the current study, we assessed interspecies sequence conservation level and proximity to some genetic elements as indicators of the potential relevance of a microsatellite to the posttranscriptional functional integrity of the corresponding gene. Interspecies sequence conservation level and proximity to other genetic elements were not associated with microsatellite length variation. The presence of flanking putative miR binding sites initially appeared to be associated with higher somatic MSI rates. This association became undetectable in comparisons of microsatellites of the same length, although the number of loci in each comparison was limited. These observations indicate that functional relevance of microsatellite is not the major determinant of length polymorphism in both MMR-deficient and -proficient settings in human.

An exceptionally high incidence of tumors possessing mutations in a gene suggests that cells containing these mutations were subjected to clonal expansion in multiple individuals due to certain selective advantages provided by these mutations during multi-step carcinogenesis [26]. In the current study, we identified three 3′UTR microsatellite loci that demonstrated high mutation rates in primary MSI-H colorectal tumors, even when this microsatellite length-dependent mutability in MMR-deficient cells was taken into account. These loci included the T_11_ repeat in the multi-functional gene, *RB1CC1* (also known as *FIP200*), the A_10_ repeat in the pro-survival and anti-apoptotic serine-threonine kinase gene, *NUAK1*, and the T_10_ repeat in the PAF1-complex subunit gene, *RTF1*, that is essential to histone methylation and Notch signaling [27], [28], [29]. Mutations in these microsatellites did not correlate significantly with clinical attributes of the tumors in our analysis. However, the relatively limited sample size in some tumor subgroups, may have limited detectable differences.

Of the three prevalent 3′UTR MSIs, *RB1CC1* mutation was the most likely to be functionally relevant for three reasons: 1) the observed *RB1CC1* somatic mutation rate exceeded the microsatellite length-based 99% prediction limit by the largest magnitude (1.7-fold), 2) significant *RB1CC1* mRNA overexpression was observed in primary MSI-H CRCs relative to nonneoplastic colonic mucosae, and 3) *RB1CC1* 3′UTR MSI was predicted to alter the secondary structure of putative binding sites for miRs-133 and -138. One of miR functions is promotion of mRNA decay by directing polyA tail removal via incomplete complementary binding to mRNA [30]. Kertesz *et al.* showed that changes in target mRNA secondary structure reduced miR effects, when the change was loss of loop structures within miR binding sites [31]. The *RB1CC1* 3′UTR microsatellite mutant was predicted to lose a large loop structure within its putative miR-138 binding site, making disruption of the miR-138-*RB1CC1* interaction and subsequent posttranscriptional downregulation likely in the mutant. The observed *RB1CC1* mRNA upregulation in RB1CC1 mutant MSI-H CRCs is consistent with this hypothesis. Further direct assessments are needed to investigate this potential scenario.

RB1CC1 plays complex roles in cellular pathways relevant to carcinogenesis [27]. RB1CC1 acts oncogenic in promoting cell survival and migration by activating Wnt signaling, TNF-alpha-induced JNK activity, and mTOR signaling *in vitro* and *in vivo* [32], [33]. RB1CC1 acts tumor suppressive in inhibiting cell cycle progression and proliferation by RB1 induction, p53 stabilization, cyclin D1 destabilization, suppression of PyK2 and FAK, and STAT protein inhibition *in vitro* [34], [35], [36], [37], [38]. Despite the frequent *RB1CC1* deletion in primary human breast cancers, RB1CC1 conditional knockout in mamillary gland and skin failed to promote tumorigenesis [39], [40]. These reports indicate that the impact of RB1CC1 upon tumorigenesis differs depending upon context. The mRNA upregulation and relative rarity of *RB1CC1* biallelic mutation observed in MSI-H colorectal tumors appears to support potential oncogenic roles of RB1CC1 in the colon. Consistent to this notion, the *RB1CC1* locus (8q11.23) is frequently amplified in CRCs [41]. A putative RB1CC1-targeting microRNA, *miR-138*, is a tumor-suppressor miR and downregulated in non-colonic malignancies [42], [43]. Taken together, it is speculated that RB1CC1 may act as an oncogene in large intestine, and that 3′UTR MSI may serve as an upregulatory mechanism in place of genomic amplification in MSI-H cancers that typically lack chromosomal aberrations.

In summary, we verified the strong dependency of MSI incidence upon microsatellite length in MSI-H colorectal tumors and, to a lesser degree, in MMR-proficient cells by conducting an extensive survey of well-characterized short 3′UTR microsatellites. In contrast, relevance of a microsatellite to its corresponding gene functionality appeared to have little impact upon MSI incidence. We also established a lack of correlation between MSI incidence and sequence conservation level or distance from genetic elements. Our data indicate that microsatellite length dependency should be taken into account when evaluating 3′UTR MSI in MMR-deficient cells. This study also provides a screening strategy for novel functionally relevant 3′UTR MSI events in human tumorigenesis. This screening identified frequent mutation and aberrant mRNA upregulation of *RB1CC1* in MSI-H colorectal tumors, which deserves further investigation of its potential involvement in colorectal tumorigenesis.

## Supporting Information

Table S1 Description of 3′UTR microsatellites analyzed in the current study.
(0.04 MB XLS)

Table S2 Germline 3′UTR microsatellite variations observed in the current study.
(0.02 MB XLS)

Table S3 Tumor attribute-dependent 3′UTR microsatellite mutational profiles.
(0.02 MB XLS)
